# Comparison among Amber-ff14SB, Amber-ff19SB, and CHARMM36 Force Fields for Ionic and Electroosmotic Flows in Biological Nanopores

**DOI:** 10.1021/acs.jctc.5c01032

**Published:** 2025-11-13

**Authors:** Simone Gargano, Domingo Francesco Iacoviello, Federico Iacovelli, Blasco Morozzo della Rocca, Mauro Chinappi

**Affiliations:** † Department of Industrial Engineering, 9318 University of Rome Tor Vergata, Via del Politecnico 1, 00133 Roma, Italy; ‡ Department of Biology, University of Rome Tor Vergata, Via della Ricerca Scientifica 1, 00133 Roma, Italy

## Abstract

Molecular dynamics has been widely used in nanofluidics as it provides an atomistic description of fluid transport that cannot be achieved experimentally. The interactions among atoms are modeled by empirical force fields whose reliability for a specific problem must be tested against experimental evidence. Such validation is often difficult to obtain in nanofluidics due to the challenges associated with measuring nanoscale flows. Here, we systematically compare three popular force fields (CHARMM36, Amber-ff14SB, both with TIP3P water, and Amber-ff19SB with the OPC water) in terms of their electrohydrodynamical outputs. As test cases, we used CytK and MspA, two biological nanopores employed in sensing. For each nanopore, we simulated one cation-selective and one anion-selective mutant. Our results show that while, overall, the anion/cation selectivity and the direction of electroosmosis are coherent across the force fields, quantitative differences are observed. These differences cannot be simply explained by the transport properties of the solution, such as the ion conductivity and viscosity. For narrow pores such as MspA, these differences can become so significant as to yield qualitatively different outcomes; for example, one force field may predict no detectable electroosmosis while another shows a clear electroosmotic flow.

## Graphical abstract

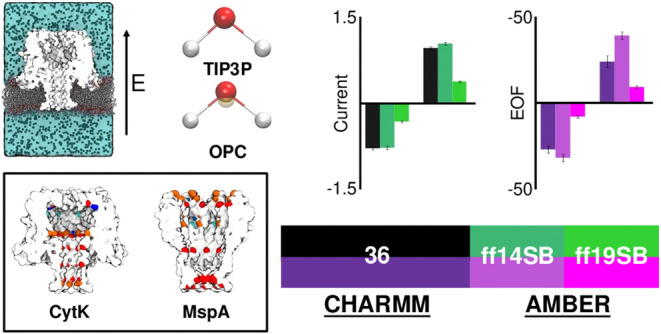

## 1 Introduction

Molecular Dynamics (MD) simulations have become essential tools for investigating biophysical phenomena, providing insights into their underlying mechanisms at the atomic level.^1–6^ In recent decades, several works have employed MD to study the transport across biological and solid-state pores, such as ion conductance,^7,8^ wetting/dewetting dynamics,^9,10^ and motion of biomolecules.^11–16^ Nonequilibrium MD has also been used to model the electroosmotic flow (EOF) through nanopores. EOF is the water movement induced by an external electric field. A common mechanism leading to EOF is the gathering of counterions near charged surfaces, which, under the action of an external electric field, drag the water molecules along.^17,18^ EOF is particularly relevant in single-molecule nanopore sensing,^19^ as it allows to capture molecules regardless of their net charge.^11,20–27^ Computational prediction of EOF in nanopores is pivotal, since direct experimental techniques to measure EOF in narrow biological nanopores are not feasible and indirect estimations via selectivity measurements are debated.^28^ In MD, interaction models and simulation setups are critical for accurately reproducing the phenomena of interest. A key component is the choice of force fields, which are the models for the potential energy governing the interatomic interaction.^29^ Among the most widely used force fields are CHARMM (with its latest version, CHARMM36 ^30^) and Amber-based force fields (such as ff14SB^31^ and ff19SB^32^). Another essential ingredient for achieving realistic MD simulations is the water model. Over the past four decades, numerous water models have been developed to better approximate the physical and chemical properties of water observed experimentally.^33,34^ The Transferable Intermolecular Potential with 3 Points (TIP3P) model,^35^ which features three interaction sites, is the standard water model for the CHARMM36 force field (although usually a modified version is used^36^). TIP3P is also compatible with the Amber-ff14SB force field. One of the most recent water models is the Optimal Point Charge (OPC),^37^ a four-site model, where a dummy atom is added near the oxygen atom along the bisector of the HOH angle. Among available water models, OPC is the most suitable choice for the Amber-ff19SB force field.^32^ Concerning ion currents and EOF, the most relevant difference between OPC and TIP3P is undoubtedly the viscosity, that is 2.5-fold higher for OPC than for TIP3P, and more similar to experimental water.^38,39^

Several studies explored the role of force fields.^40,41^ For instance, different AMBER and CHARMM protein force fields yield variations in simulating protein folding and the dynamics of disordered proteins, impacting whether simulated ensembles accurately match experimental data such as NMR measurements.^41^ Similarly, the force field choice influences the simulation of protein–protein interactions, affecting protein complex stability and the nature of the interaction interface.^42^ The force field also dictates the accuracy of representing specific protein structural properties and conformational ensembles; however, improvements in reproducing one structural property when changing force field, often result in decreased accuracy in another feature.^43^ The water model is equally critical. Explicit water models are generally preferred for their detailed treatment of hydration, as opposed to implicit solvent ones. However, the specific choice among models like TIP3P,^35^ SPC/E,^44^ TIP4P variants,^45^ or OPC^37^ may significantly affect simulation outcomes. For instance, it has been shown that water models can lead to different predictions of conserved water molecules in binding sites and impact the stability of protein-glycan complexes.^46^

In this study, we focused on two biological nanopores: CytK and MspA. CytK is a β-barrel nanopore found in *Bacillus cereus*, previously used in nanopore sensing.^11,47^ We employed two mutants, CytK-2E4D and CytK-6K, characterized by cationic and anionic selectivity, respectively.^11^ MspA, an octameric porin from *Mycobacterium smegmatis*, is another popular nanopore in the sensing community.^48–53^ Here, we studied the wild-type (MspA-WT), that is cation-selective, and its M3 mutant,^54,55^ the latter is engineered with mutations that remove negative residues from the constriction and add positive ones to make the pore anion-selective. For each nanopore, we performed nonequilibrium MD simulations with three different force fields (CHARMM36, Amber-ff14SB, both with TIP3P, and Amber-ff19SB with OPC) to measure ionic current and EOF. The main result of our study is that, while the selectivity and the direction of EOF are, in most cases, consistent across the three force fields, quantitative differences are observed. Some of them are expected from the known differences in the viscosity of the water model used in our study, while others suggest that the ion-protein interaction significantly affects anion and cation distribution, resulting in relevant differences in the ionic and electroosmotic current that cannot be simply explained by the conductivities and viscosities of the ion and water models, respectively.

## 2 Methods

### 2.1 Molecular Dynamics Simulation

All of the MD runs were carried out using NAMD 2.14 ^57^ with a time step Δ*t* = 2.0 fs (unless otherwise stated) and the particle mesh Ewald^58^ method with a 1.0 Å spaced grid for long-range electrostatic interactions. A cutoff of 12 Å (switching distance of 10 Å) was used for the short-range nonbonded interactions, with 1–4 scaling applied. This scaling parameter was set to 1.0 for CHARMM36-based^30^ simulations and 0.833333 for Amber-based^59^ simulations. Periodic boundary conditions with an orthogonal box are used. A Langevin thermostat was used for all of the simulations while a Nosé–Hoover Langevin piston pressure control was employed for constant pressure simulations.^60^ CUFIX corrections^61,62^ for ions were applied for CHARMM36 ^30^ and Amber-ff14SB^31^ simulations. Amber-ff14SB simulations without CUFIX corrections were also performed for comparison.

### 2.2 Conductivity Measurements

The water box (80 Å × 80 Å × 80 Å) used for conductivity measurements was generated by VMD solvate package^63^ while K^+^ and Cl^–^ ions were added using the VMD autoionize package. The resulting pdb and psf were employed for the simulation using the CHARMM36 force field^64^ with modified TIP3P^36^ water model. For Amber-ff14SB^31^ and Amber-ff19SB^32^ simulations (with TIP3P^35^ and OPC^37^ water models, respectively), additional steps were necessary to produce a prmtop file and to convert the CHARMM36-formatted pdb file in an Amber-formatted pdb file. Details of the conversion script and related materials are provided in the Supporting Information and in an accompanying downloadable folder. After 5000 steps of energy minimization, the system was equilibrated by a 0.1 ns NPT run (Δ*t* = 1.0 fs). Nonequilibrium simulations were run under an external electrical field **E** = (0, 0, *E*_*z*_) with

1 $$E_{z}=\frac{\Delta V}{L_{z}}$$

where Δ*V* is the applied voltage and *L*_*z*_ is the size of the box along the z-axis. A thermostat was applied to the oxygen atoms of the water. For each Δ*V*, we ran 50 ns, with the first 10 ns being discarded in the analysis. The electric current *I* was measured as it follows.^8,65^ During the production run, trajectories were printed every τ = 20 ps. In postprocessing, we calculated^8,65^

2 $$I\left(t\right)=\frac{1}{\tau L_{z}}\sum_{i=1}^{N}q_{i}\left[z_{i}\left(t+\tau \right)-z_{i}\left(t\right)\right]$$

where *I*(*t*) is the average current in the interval *t* ∈ [*t*, *t* + τ], *q*_*i*_ is the charge of the *i*th ion, *z*_*i*_ its *z*-coordinate, and *N* the number of ions in the system. *I* is estimated averaging the current time trace from eq 2, while errors are calculated using a block average protocol, with a block size of 10 ns. Cation and anion contributions to *I* were calculated, restricting the summation in eq 2 to only K^+^ and Cl^–^.

### 2.3 Nanopore and Membrane Setup and Equilibration

The protocol used to build and equilibrate the system is similar to the one reported in Di Muccio et al.^8^ which is also similar to the one used in previous works.^7,68^ We used the same setup (Figure 1) for all of the nanopores. For CytK-2E4D, we used the same structure employed in Sauciuc et al.^11^ while for MspA-WT, we used the X-ray structure PDB_ID: 1UUN^69^ taken from the OPM database,^70^ where arginine 96 was mutated to alanine to restore the WT sequence. Mutations to obtain CytK-6K and MspA-M3 were performed using the Mutator Plugin of VMD.^63^ The pore axis was aligned with the *z*-axis of the reference system. The pore was inserted in a POPC bilayer membrane generated using VMD.^63^ Lipid molecules overlapping with the nanopore or located in the pore lumen were removed. Subsequently, water and ions were added using the solvate and ionize VMD plugins.^63^ After 5000 steps of energy minimization, a first 494 ps NPT equilibration step (*P* = 1 atm, flexible cell, constant ratio, *T* = 300 K, and time step Δ*t* = 0.5 fs) was performed. In this first step, external forces were applied to the water molecules to avoid their penetration into the membrane. The *C*_α_ atoms of the nanopore were kept fixed, and the phosphorus atoms of the lipid heads were harmonically constrained to their z-positions (spring constant *k* = 1 kcal/mol·Å textsuperscript2). An initial temperature ramp was imposed (every 5 ps, the velocities are rescaled to a temperature of 0, 25, 50, ···, 275, 300 K) for a smooth relaxation of the system. In essence, in this step, the lipid tails and the electrolyte relax while the nanopore backbone is fixed. In a second, 1 ns long (Δ*t* = 1 fs) NPT equilibration step, all of the constraints on the membrane and the protein were removed. All of the other control settings were as in the previous step. The final equilibration step was an NPT run of 1 ns (time step 1 fs) with no constraints on the lipids and no external forces to keep the water molecules out of the membrane. At the end of the equilibration procedure, the three dimension measures of the box were the following: for CytK nanopores, *L*_*x*_ = *L*_*y*_ ≃138 Å, and *L*_*z*_ ≃186 Å, and a total of about 359 K atoms, for MspA, *L*_*x*_ = *L*_*y*_ ≃130 Å, and *L*_*z*_ ≃195 Å, and a total of about 333 K atoms. Subsequently, nonequilibrium simulations were run with an external electrical field **E** = (0, 0, *E*_*z*_), eq 1. Even though, for nonhomogeneous systems, the application of a uniform electric field throughout the system does not necessarily correspond to a voltage difference at the boundaries, this approach was shown to be equivalent to the application of a voltage.^18,56^ The nanopore *C*_α_ values were constrained, while the phosphorus atoms of the lipids were fixed. A thermostat was applied to protein and lipids with the exception of the constrained atoms. For each nonequilibrium simulation, we ran 40 ns, discarding the first 10 ns from the analysis. Trajectories were printed every τ = 20 ps and we calculated currents using the same protocol employed for the triperiodic water box, see eq 2. The EOF was measured similarly, computing the summation over the oxygen atoms of the water molecules and counting the molecules. For each Δ*V*, we ran different replicas of the above simulation protocol. For all of the CytK simulations and for Amber simulations for MspA, the protocol was repeated starting from the equilibration. For each replica, average currents and EOF were calculated, and the final estimation of the current was obtained by averaging the results for different replicas, while its error was estimated by dividing the standard deviation of the averages by the square root of the number of replicas. For the CHARMM36 MspA nanopores, a slightly different protocol was used. We ran a single equilibration, and then, starting from it, several replicas were run in parallel to increase statistics.

**Figure 1:**
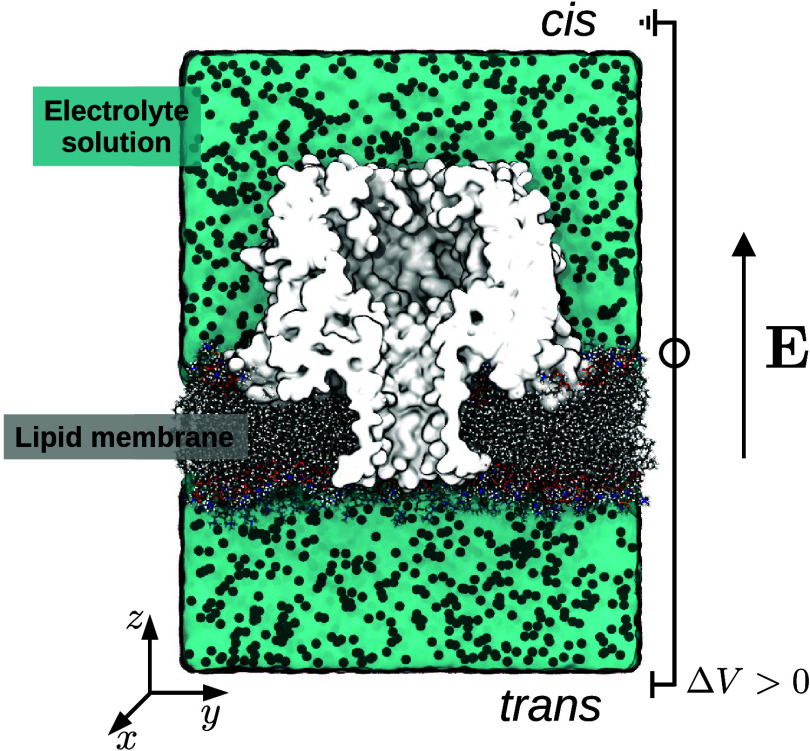
System setup. The nanopore was embedded in a lipid membrane and immersed in a 1 M KCl solution. Periodic boundary conditions were used in all directions. An electric field was applied along the pore axis (z-direction). This corresponds to a voltage drop Δ*V* between the cis and trans reservoirs.^56^ The figure refers to the CytK-2E4D nanopore. A similar setup was used for all other cases.

### 2.4 Ion and Water Density Fields

Following a procedure presented in our previous works,^8,50,68^ the box was initially divided into cubic cells with a side length of 1 Å. The average density for water, cations, and anions in each cell was calculated using the “Volmap” plugin of VMD.^63^ The 3D density fields were then transformed from a Cartesian coordinate system to a cylindrical coordinate system with respect to the nanopore axis. Finally, the average on the angular coordinate allowed us to obtain the (*r*, *z*) maps represented in the manuscript. To evaluate possible systematic differences among the simulations with the three force fields, we set up the following procedure. For each pore, we selected a region of interest (ROI) in the (*r*, *z*) map that contains the area with the most relevant charge accumulation, i.e., the barrel for CytK and the constriction for MspA. Specifically, for CytK mutants, a 40 Å × 70 Å ROI centered at *r* = 0 Å, *z* = 15 Å was chosen, while an ROI of 40 Å × 50 Å, centered at the origin, was chosen for MspA nanopores. Then, given two maps corresponding to the same pore but to different replicas or force fields, we calculated their distance *d*_*ij*_ as

3 $$d_{\mathrm{ij}}^{2}=\int_{\mathrm{ROI}}{\left[f_{i}\left(r,z\right)-f_{j}\left(r,z\right)\right]}^{2}drdz$$

where *f*_*i*_ and *f*_*j*_ are the densities for the *i*th and *j*th maps. Then, we calculated the average of *d*_*ij*_ when *i* and *j* are replicas using the same force field (*intra*-group comparison) and when *i* and *j* belong to the same system with different force fields (*inter*-group comparison). The relation between the *intra*- and *inter*-group comparison provides an indication of possible systematic differences among the ion and water distributions obtained with different force fields.

## 3 Results and Discussion

### 3.1 Electrolyte Conductivity

As a preliminary step, we evaluated the conductivity of KCl for three different force field/water model combinations, specifically: CHARMM36 and Amber coupled with TIP3P water and Amber force field with OPC water (see Section 2), at varying salt concentrations. Note that, for Amber, the parameters for ions are not included in the ff14SB and ff19SB frameworks. Consequently, it is not appropriate to refer to these simulations as Amber-ff14SB and Amber-ff19SB but just as Amber with TIP3P and Amber with OPC. The ion parameters were taken from^71^ and adapted to each water model, as suggested by the Amber user guide. For TIP3P simulations, CUFIX corrections^61^ were employed, while for OPC no corrections were applied. We used a triperiodic box with electric field applied along the *z*-axis, corresponding to a voltage Δ*V* = *E*_*z*_ *L*_*z*_ with *L*_*z*_ the box size in the *z*-direction, eq 1. For each Δ*V*, we measured the current *I*. The conductivity was estimated as

4 $$\sigma =G\frac{L_{z}}{A}$$

where *G* is the conductance and *A* = *L*_*x*_ *L*_*y*_ the area perpendicular to the *z*-axis, see Figure S1 of the Supporting Information for current–voltage curves and for further information on conductivity calculation and error estimation. Figure 2b shows a clear difference in σ among the three force field/water model combinations. More specifically, the two TIP3P cases (CHARMM36 and Amber) provide very similar conductivity for all KCl concentrations, while the OPC case results in a *ca.* two times smaller conductivity. This difference can be partially explained by the different viscosities of the two water models. Indeed, the TIP3P and OPC exhibit viscosities of 0.32 and 0.80 mPa·s, respectively.^38,39^ For comparison, in Figure 2b, we reported previous MD estimations of σ by Aksimentiev et al.^7^ and Modi et al.,^67^ both obtained with CHARMM27 ^72^ coupled with TIP3P water, highlighting the importance of the ion model in the estimation of currents. We also reported experimental data by Pezeshki et al.,^66^ noting that it is closer to TIP3P simulations, despite OPC having a viscosity closer to real water, which is 0.90 mPa·s. Additionally, we reported cationic and anionic contributions to the current (Figure 2c), showing that the two contributions are similar, in line with the similar mobility of the two ions.^17^

**Figure 2:**
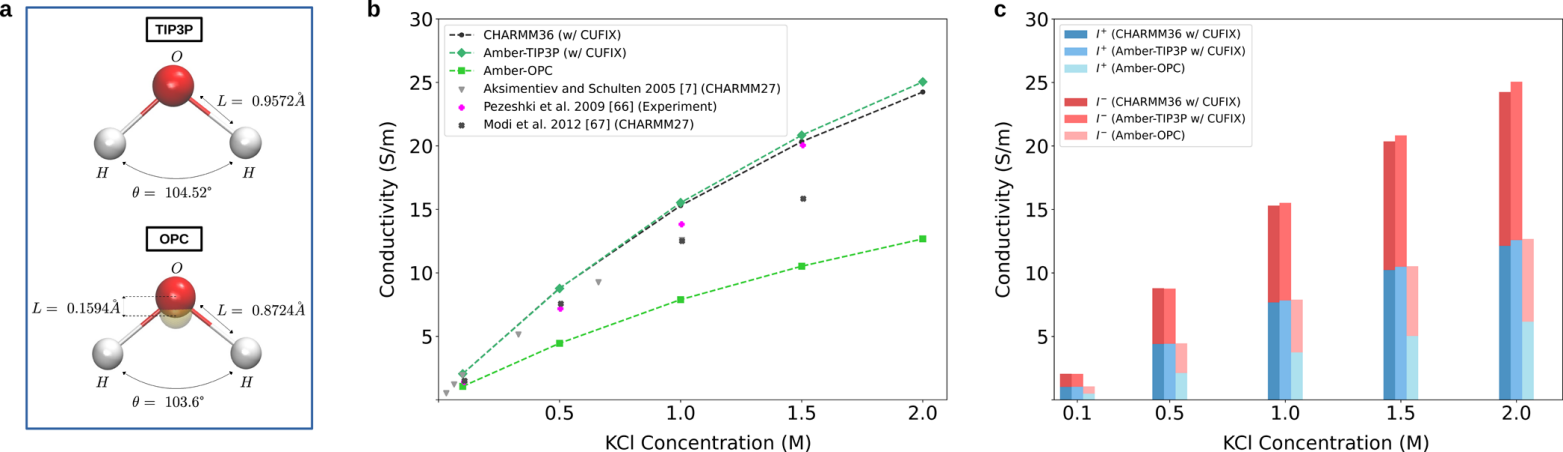
Comparison of electrolyte conductivities. (a) Water models used in the simulations. (b) Conductivity as a function of KCl concentration. Lines refer to the data reported in the present manuscript. Literature data, both from MD simulations and from experimental assays, are reported for comparison as unconnected data points.^7,66,67^ (c) K^+^ and Cl^–^ contributions to conductivity. Errors in conductivities are smaller than the size of the marker symbol. The conductivity is calculated from current–voltage curves as detailed in Supporting Figure S1.

### 3.2 EOF and Ionic Currents through Nanopores

We then evaluated the ion selectivity and EOF of four nanopores: two mutants of CytK and two mutants of MspA. The simulation setup is reported in Figure 1. An electric field *E*_*z*_ = Δ*V*/*L*_*z*_ was applied along the *z*-axis, where Δ*V* is the voltage applied at the trans side.

CytK-2E4D contains four negatively charged aspartic acids (D128-D145-D151-D155) and two negatively charged glutamic acids (E112-E139) exposed to the pore lumen (Figure 3a). Instead, CytK-6K incorporates six positively charged lysines in the same locations (K112-K128-K139-K145-K151-K155) (Figure 3f). CytK-2E4D is selective for cations, as is evident from the higher contribution of the cationic current with respect to the anionic one, as expected from the negative residues in the pore lumen (Figure 3b–d). Conversely, CytK-6K displays anion selectivity consistent with the positive surface charges conferred by the lysines (Figure 3g–i). To corroborate this interpretation, ion concentration maps at Δ*V* = 0 are reported. For CytK-2E4D, the maps show a high concentration of cations and a depletion of anions in the region close to the charged residues (Figure 3a). Instead, CytK-6K displays a higher density of anions (Figure 3f). The direction of the EOF for both pores is consistent with the movement of the counterions for both positive and negative voltages. For instance, at Δ*V* = 125 mV, the cation-selective CytK-2E4D has positive EOF (i.e., directed from trans to cis, Figure 3e), while the anion-selective CytK-6K (Figure 3j) has a negative EOF. The comparison among the three force fields indicates that Amber-ff19SB with OPC water provides the smallest values for both EOF and ionic currents. CHARMM36 and Amber-ff14SB provide estimates of current and EOF that are quite similar for CytK-2E4D. Instead, some significant differences are evident for CytK-6K, in particular, ionic current and EOF are larger for the Amber-ff14SB force field at negative voltages.

**Figure 3:**
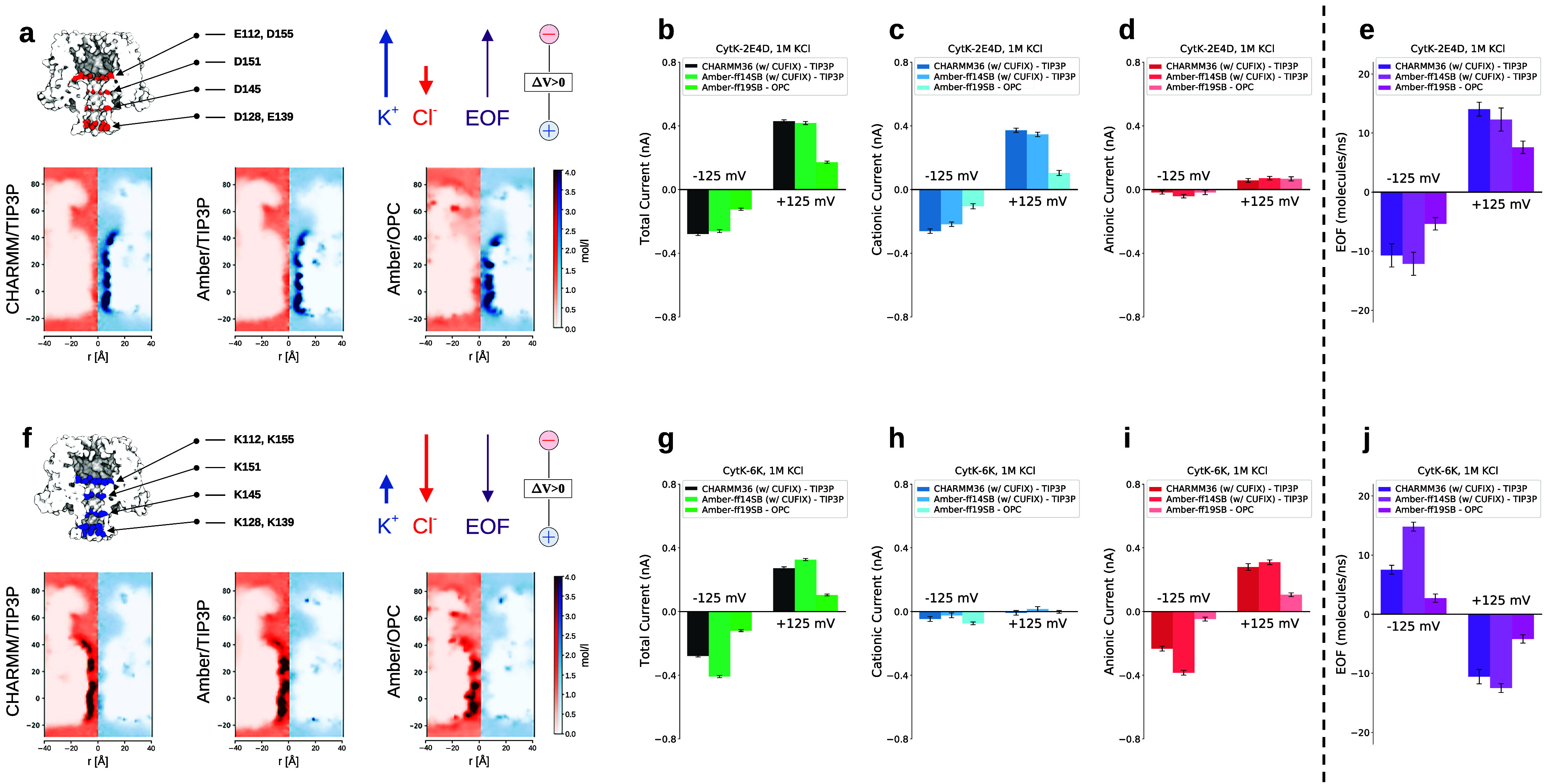
CytK nanopores: ionic current and EOF at KCl 1M. (a) Sketch of CytK-2E4D with the most relevant acidic residues colored in red and ion concentration maps (Cl^–^ in red, K^+^ in blue). (b–d) Total, cationic (K^+^), and anionic (Cl^–^) currents. (e) EOF. (f–j) Same data as in panels (a–e) but for CytK-6K. In panel (f), the most relevant positively charged residues are shown in blue. Error bars refer to standard errors calculated over ten independent replicas. Detailed representations of all charged residues exposed toward the pore lumen are shown in Supporting Figure S2.

We then repeated the analysis for MspA. MspA-WT contains several charged residues exposed toward the pore lumen, as shown in Supporting Figure S3. The residues most relevant for selectivity are the three negatively charged aspartic acids in the constriction region (D90-D91-D93, Figure 4a) and the pore is expected to be cation selective. However, in the mutant MspA-M3, the constriction is uncharged (D90N-D91N-D93N), and arginines are added in positions R88-R96-R116; thus, the pore is expected to be anion selective (Figure 4f). Sequences and the distributions of charged residues in the pore lumen can be found in Supporting Figure S3.

**Figure 4:**
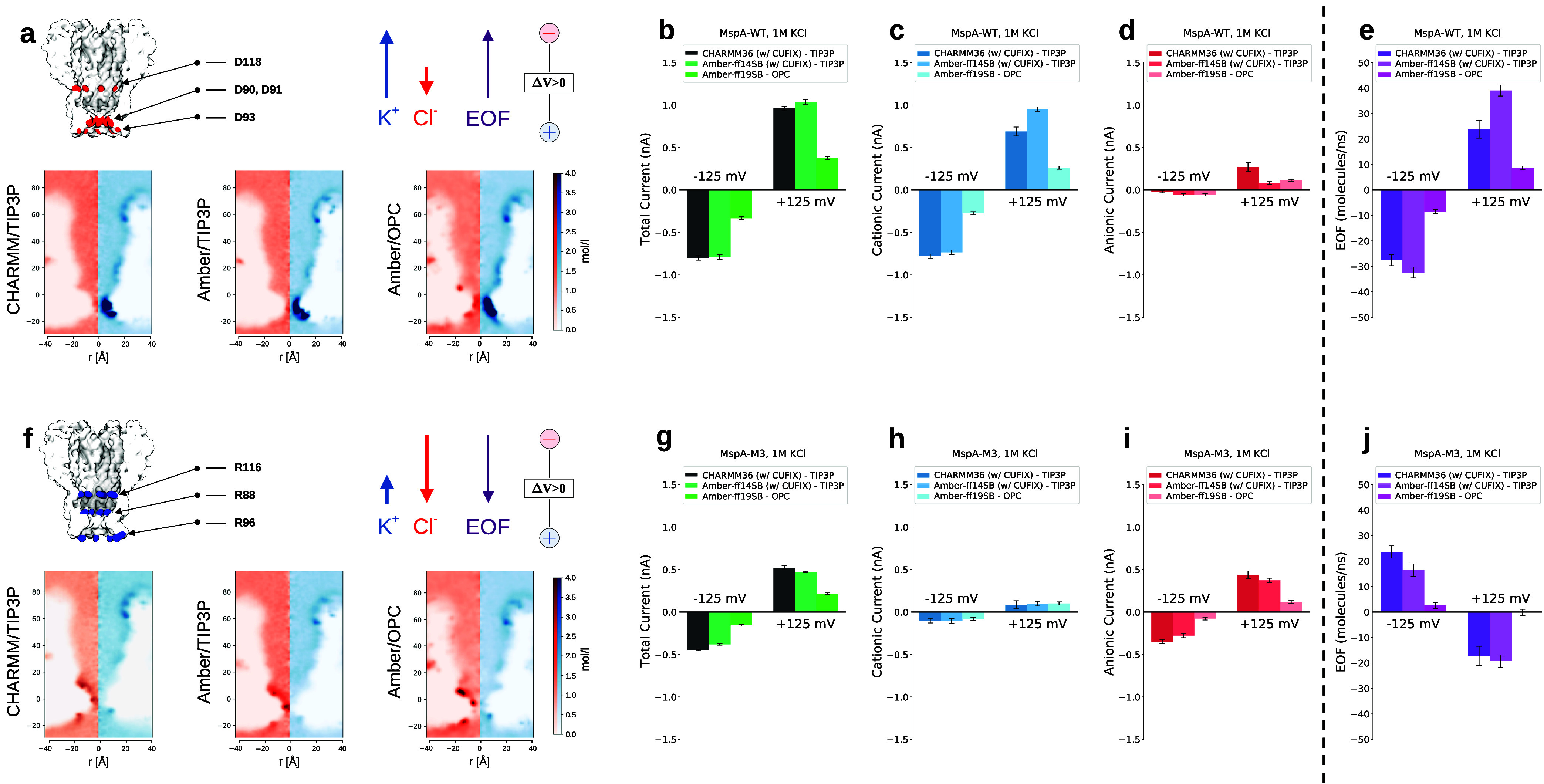
MspA nanopores: ionic current and EOF at KCl 1M. (a) Sketch of MspA-WT with the most relevant acidic residues colored in red and ion concentration maps (Cl^–^ in red, K^+^ in blue). (b–d) Total, cationic (K^+^), and anionic (Cl^–^) currents. (e) EOF. (f–j) Same data as in panels (a–e) but for MspA-M3. In panel (f), the most relevant positive residues are in blue. Error bars refer to standard errors calculated over ten independent replicas. Detailed representations of all of the charged residues exposed toward the pore lumen are shown in Supporting Figure S3.

As for CytK, the overall result is that one pore is cation selective, while the other is anion selective, and that EOF is consistent with the flow of counterions; i.e., in MspA-WT (cation selective), the EOF goes in the direction of cations (Figure 4b–e,4g–j). Overall, CHARMM36 and Amber-ff14SB show similar results, with the exception of MspA-WT at Δ*V* = 125 mV. The cationic current and the EOF are larger for Amber-ff14SB compared to CHARMM36. Remarkably, the OPC produces qualitatively different results. In fact, selectivity and EOF are not observed in MspA-M3 at Δ*V* = 125 mV and are extremely low for Δ*V* = −125 mV. This contrasts with the results for the other two force fields. For comparison, we also ran simulations with Amber-ff14SB without CUFIX correction for both CytK and MspA nanopores; see Supporting Figures S4–S5. For CytK, ionic currents are not affected by CUFIX correction, while EOF for positive voltages shows a slight difference. A more pronounced difference is apparent for MspA, in particular, for the wild-type. This is presumably due to the high concentration of cations present in the constriction of MspA-WT that makes the role of the ion force field details more relevant.

The above-mentioned findings indicate that the differences in the flow are not just proportional to the known differences in the viscosity of the TIP3P and OPC water models (a factor *ca.*2.5); otherwise, CHARMM36-TIP3P and Amber-ff14SB would always produce very similar currents. To better highlight this phenomenon, in Figure 5, we report the currents rescaled with respect to the CHARMM36 case. More specifically, we rescaled EOF as

5 $${\mathrm{EOF}}_{x,r}=\frac{\mu_{x}}{\mu_{\mathrm{TIP}3P}}{\mathrm{EOF}}_{x}$$

where EOF_*x*,*r*_ is the rescaled EOF for a given force field *x*, EOF_*x*_ is the EOF already reported in Figures 3 and 4, μ_*x*_ is the viscosity of the corresponding water model and μ_TIP3P_ the viscosity of TIP3P water. Similarly, we rescaled the currents with the respective conductivities, i.e.,

6 $$I_{x,r}=\frac{\sigma_{\mathrm{CHARMM}36}}{\sigma_{x}}I_{x}$$

where *I*_*x*,*r*_ is the rescaled current for a given force field *x*, σ_*x*_ is the conductivity of the corresponding force field at 1 M KCl and σ_CHARMM36_ is the conductivity of TIP3P water. These representations better highlight that the differences between the ionic currents and EOF among the force fields analyzed do not simply scale linearly with transport properties of the solution such as conductivity and viscosity. These differences may originate from subtle variations in ion-protein or ion–water interactions that are specific to each force field and affect the motion of the ions and their local distribution near the pore constriction.

**Figure 5:**
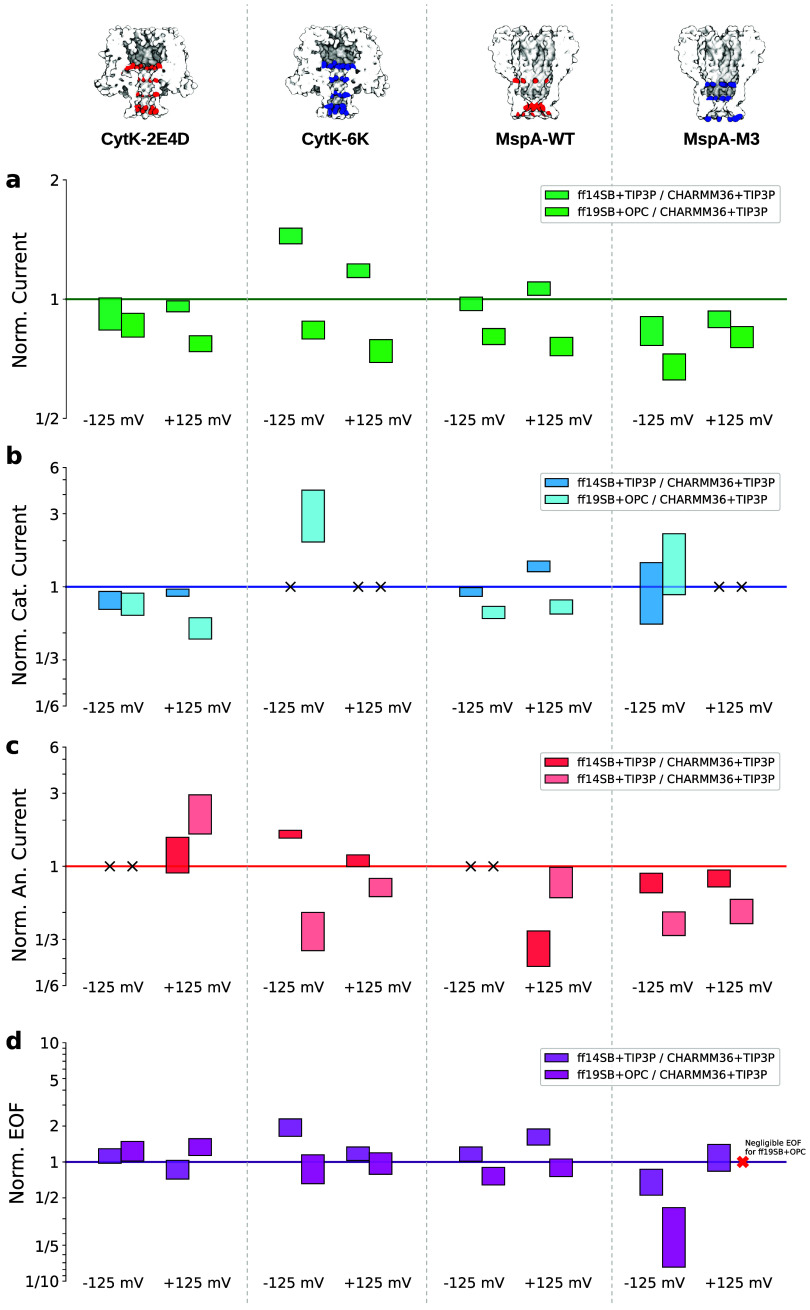
Rescaled currents and EOF. (a–c) Ionic currents rescaled with the CHARMM36 values and conductivities, eq 6. (d) EOF rescaled with CHARMM36 value and viscosities, eq 5. The bars correspond to the average value ± statistical error. Deviations from the horizontal line (rescaled values equal to 1) indicate that the differences among the force fields are not simply related to conductivity and viscosity. Rescaled EOF for MspA-M3 at Δ*V* = 125 mV for Amber-ff19SB with OPC (red cross) is not reported since it is negligible; specifically, the EOF for Amber-ff19SB is almost zero while for CHARMM36 it is ≃ 20 molecules/ns, see Figure 4. Cases marked with black crosses are not reported, as both the numerator and the denominator are almost negligible, i.e., statistical error smaller than half of the average current; consequently, the propagated statistical error on the ratio is extremely large.

To test this hypothesis, we compared the ion density maps. Our data set for CytK nanopores consists of ten replicas for each force field. Hence, for a given voltage, nanopore and ion (K^+^, Cl^–^), we need to compare 3 × 10 maps. Due to the slightly different protocol used for MspA (see Methods), in this case, we have ten independent maps only for Amber-ff14SB and Amber-ff19SB while for CHARMM36, we have a single map. We first investigated whether the variability among the ten replicas for a given force field (*intra-*group comparison) is larger than the variability among groups (*inter*-group comparison). The metric selected for the comparison is the square root of the integral of the square of the difference of two maps; see eq 3 in Methods. Figure 6a–b reports the average of the *intra* and *inter* distances, at Δ*V* = 125 mV, for anions and cations. It is evident that the *intra*-group variability is, in general, smaller than the *inter*-group variability. For instance, indicating for brevity with CT the CHARMM36 with TIP3P water and with AT and AO the Amber-ff14SB with TIP3P and the Amber-ff19SB with OPC, we have that the CT-CT map distance (*intra*) for K^+^ and CytK-2E4D is around 18 while the *inter*-group comparisons CT-AT, CT-AO, and AT-AO are all larger than 30. This indicates that as hypothesized from the rescaled currents in Figure 5, the difference in the force field results in a different ion distribution. Similar conclusions can be drawn for other pores, although, in some cases, the *inter* and *intra* differences are not large; see e.g., Cl^–^ for CytK-2E4D. As a general trend, the differences are more marked for the counterions. Figure 6c–d reports some examples of the difference in ion distribution. In particular, for the CytK-6K case at Δ*V* = – 125 mV, cations are almost absent in the barrel for the Amber-ff14SB and, coherently, the anion clouds are slightly more intense. This may explain the larger ionic current observed for Amber-ff14SB, see Figure 3i. Data for additional cases are reported in Supporting Figures S7–S8, confirming the scenario described scenario. One potential source of variability is the different configurations that the proteins adopt during the simulations. To quantify possible differences in the protein structure, we used as metric the RMSD calculated on the residues in the barrel (for CytK) and in the constriction (for MspA). No systematic differences are observed; see Supporting Figure S9. These findings suggest that the crucial difference in the force field is the interaction between the protein and the ions that results in a slightly different ion distribution. When these differences are localized in the narrower section of the nanopore, they may give rise to relevant differences in the ionic and electroosmotic currents, which cannot be simply explained in terms of different conductivity and viscosity.

**Figure 6:**
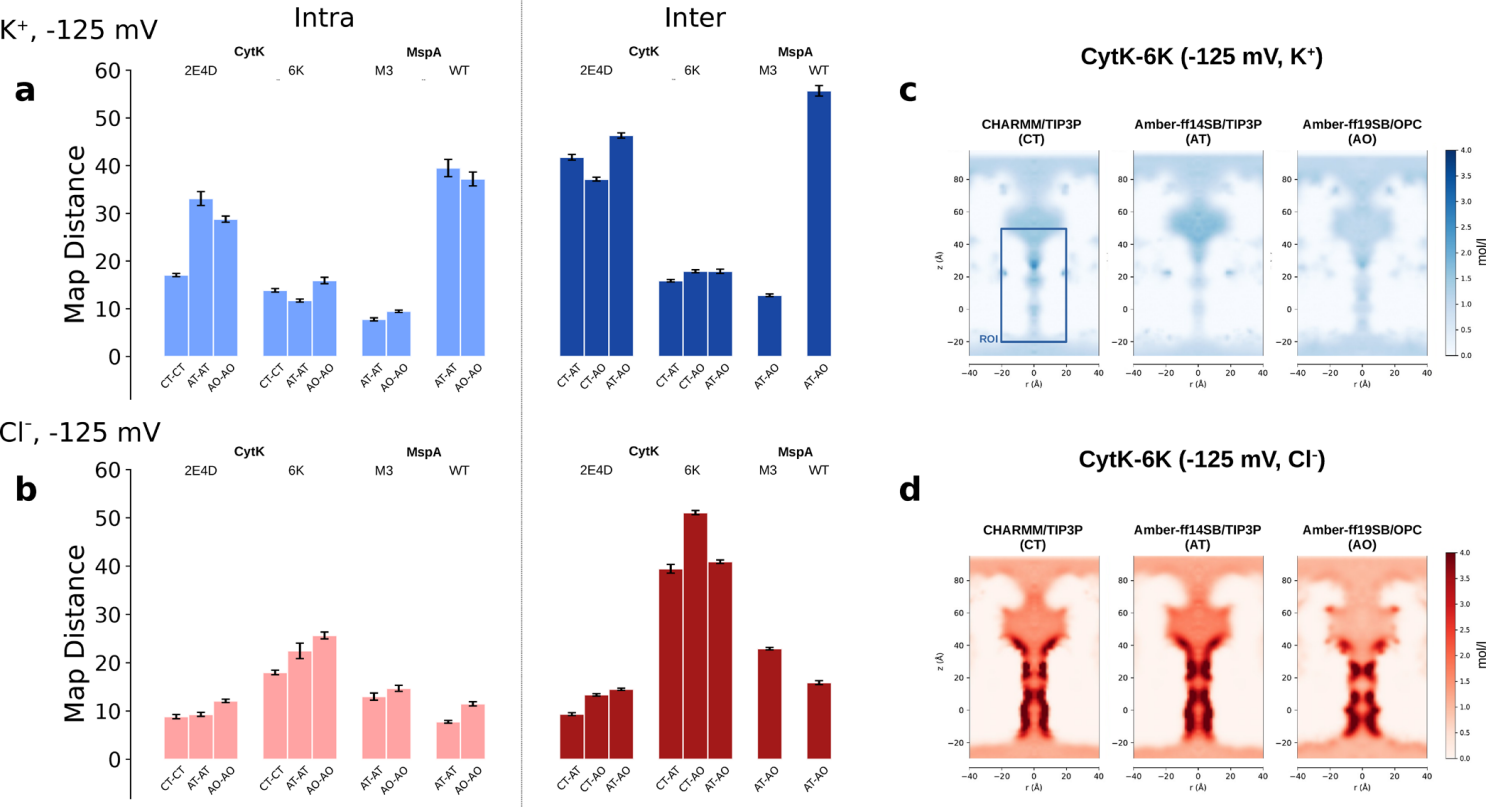
*Inter*- and *intra*-variability of ion density distributions. (a) Average distance for Cl^–^ density maps at Δ*V* = −125 mV. The distance between two force fields is calculated using eq 3 on the ROI indicated by the rectangle in panel (c). Each bar represents the average over the different comparisons of two sets. For instance, the bar labeled as CT-CT corresponds to the average of all-against-all comparisons between the ten CHARMM36 with TIP3P water (45 comparisons, replica 1 against replicas 2–10, replica 2 against 3–10, and so on), while AO-CT refers to the comparisons between 10 CHARMM36 with TIP3P and 10 Amber-ff19SB with OPC water (100 comparisons). Errors are calculated by dividing the standard deviation by the square root of the number of comparisons. (b) Same data for K^+^. Analogous data for Δ*V* = +125 mV are in Supporting Figure S6. (c–d) Selected ion density distribution maps were obtained by averaging the map of each replica. Maps for other cases are in Supporting Figures S7–S8.

## 4 Discussion

Our numerical results clearly show that EOF and ionic current predictions depend on the chosen force field. These results raise the question of which force field is most suitable for the simulations of nanopore electrohydrodynamics. One possible way to address this is to compare our results with experiments for the main transport quantities: pore conductance, pore selectivity, and EOF. In Supporting Table S2, we report experimental values for pore conductance^11,48,54,69,73,74^ while a summary of our numerical results is in Supporting Table S3. Experiments show that the conductance of MspA-WT is higher than that of MspA-M3. Every force field that we used captures this trend. Quantitatively, Amber-ff14SB without CUFIX gives the closest result. This could suggest that this is the best force field. However, when also including the data for CytK pores, it is evident that no force field reproduces the conductance well for all of the pores. This inability of current force fields to match the nanopore electrohydrodynamics is not surprising. Bulk conductivities are also not reproduced, as shown in Figure 2. This is not new: Aksimentiev and Schulten^7^ already noted that the agreement observed for αHL conductance was probably due to a fortuitous cancellation of force field errors, and should not be interpreted as a sign of full accuracy, a view we share. Even without quantitative predictive power, we may ask whether a qualitative comparison with experiments can indicate that one force field is more appropriate than another. We therefore checked whether the simulated selectivities are in qualitative agreement with experimental data (Tables S2 and S3). Selectivities are usually expressed in terms of the ratio of permeabilities to cations and anion *P*_+_/*P*_–_. The permeability ratio cannot be directly measured in experiments (isolated ion fluxes are not measurable quantities), but it can be linked to the reversal potential, which is the voltage at which the current is null when the cis and trans reservoirs contain electrolyte solutions at different concentrations.^27,75^ Not all of the experimental studies on nanopores report an estimation of *P*_+_/*P*_–_, and, to the best of our understanding, *P*_+_/*P*_–_ is not available for MspA-M3. However, for the other three pores, our data agree with the experimentally measured selectivities: MspA-WT and CytK-2E4D are cation selective, while CytK-6K is anion selective. Some quantitative differences are present among the prediction of the various force fields, see Supporting Table S2, so, one can be tempted to claim that the more appropriate field is the one that best reflects the experimental selectivity. However, in our view, this conclusion would be inappropriate. First, the theoretical link between the reversal potential and the selectivity is based on the Goldman-Hodgkin-Katz (GHK) model,^76,77^ which, like any theory, has some limitations recently discussed in two interesting studies.^78,79^ Second, the estimation of the selectivity ratio may depend on the reservoir concentrations, as reported also in Gu et al.,^75^ making its quantitative evaluation nontrivial. Third, the MD estimation of *P*_+_/*P*_–_ is affected by large statistical errors that can hardly be reduced, in particular when one of the two currents is close to zero (a more statistically robust indicator of selectivity is the difference between the currents^8^). Finally, we could also compare the EOF with experiments. However, this issue is much more delicate because the EOF cannot be measured directly in biological nanopores. Moreover, the theories that link EOF to selectivity are not generally valid, as we recently showed in.^28^ Thus, only a qualitative and indirect comparison is possible. For CytK, the data agree with the EOF direction predicted by all force fields and for all applied fields. Similarly, the MspA-WT data are consistent with our predictions. For MspA-M3, our results are less clear: as noted in Figure 4j, Amber-ff19SB with OPC water predicts no EOF, while the other force fields predict a measurable EOF. Unfortunately, experimental data for MspA-M3 do not allow us to determine which prediction is correct. An experiment aimed at capturing neutral molecules (or charged molecules against electrophoresis) should provide evidence for the EOF direction.^20–22^

## 5 Conclusions

In this study, we systematically analyzed the effect of force fields on MD simulations of electroosmotic flow and ion transport in nanopores. We considered three widely used force field/water model combinations and cation- and anion-selective mutants for two biological pores (CytK and MspA) employed in nanopore sensing. While all force fields coherently reproduced the ion selectivity and the direction of the EOF, quantitative differences were evident, particularly with Amber-ff19SB/OPC, which yielded lower currents and EOF. Notably, differences in EOF and currents could not be fully explained by water viscosity and ion conductivity, suggesting the role of more subtle features of the force field. Our results support the use of MD for qualitative predictions in nanopores but call for caution when extracting quantitative conclusions without validation. In particular, since differences were more pronounced for MspA, which has a narrower constriction, our results suggest that special care is needed when simulating narrow nanopores, as ion selectivity and EOF may be strongly affected by small variations in the arrangement of the charged residues exposed in the nanopore constriction. It is therefore advisible to test different force fields/water models combination in order to evaluate the robustness of the transport predictions. Comparisons with experimental data available in the literature do not allow us to claim which is the most appropriate force field (among the ones we studied) for electrohydrodynamic simulations. Despite this occurrence, we think that the comparison is useful and should be performed more systematically in electrohydrodynamic simulations, as it may highlight the limits of current models and suggest where improvements are needed.

## Supplementary Material





## Notes

A directory containing some protocols and scripts used to build the prmtop and pdb files for simulations with Amber force fields in NAMD2 (ZIP)
Additional data and figures as *I*/*V* Curves for bulk water box, CytK and MspA sequences and charges exposed toward the lumen, data on the effect of CUFIX for Amber-ff14SB and CytK and MspA nanopores, ion distribution maps, *inter*-group and *intra*-group RMSD, experimental literature data on nanopore conductance and selectivity (PDF)
