# Supplementary material for: Comparison among Amber-ff14SB, Amber-ff19SB, and CHARMM36 Force Fields for Ionic and Electroosmotic Flows in Biological Nanopores
Source: J Chem Theory Comput. 2025 Nov 13;21(22):11772–82. doi: 10.1021/acs.jctc.5c01032 (PMC12659023; doi:10.1021/acs.jctc.5c01032)
Supplement: Supplementary file 1 [file ct5c01032_si_001.zip › GARGANO25_CHARMM2AMBER/README.pdf]

This file and the accompanying directory refer to the Supporting Information for:
Simone Gargano, Domingo Francesco Iacoviello, Federico Iacovelli, Blasco Morozzo della Rocca, Mauro Chinappi.
Comparison among Amber ff14SB, Amber ff19SB and CHARMM36 force fields for ionic and electroosmotic flows in biological nanopores. 2025.

To generate a functioning Amber-based simulation system (ff14SB/TIP3P or ff19SB/OPC) containing protein, membrane, water, and ions, follow these steps:

1. Go to CHARMM36/MEMBUILD. Inside, you will find the Build.sh script:
- Here you can change a number of parameters.
- The default settings are configured for a CytK-2E4D protein embedded in a POPC (1-palmitoyl-2-oleoyl-sn-glycero-3-phosphocholine) membrane, in a 1 M KCl solution.
- You can enable the "mutation" section, but if you do, you must also edit the mutations.txt file in the same directory (for CytK-2E4D, mutations are not required).

2. Adjust parameters as needed, then run Build.sh.

3. Wait for the process to complete, then copy the Out/filename.pdb file to ../../AMBER/CHARMM2AMBER_SCRIPT.

4. Go to the CHARMM2AMBER_SCRIPT directory and open the script.leap file:
- Here you can choose the force field/water model combination you want to use (follow the instructions inside the file to comment/uncomment the relevant lines).

5. Open charmm2amber.sh and set the paths for the CHARMM2AMBER main directory and the AMBERTOOLS main directory.

6. Once script.leap is configured, run the conversion script with the following command:

```
./charmm2amber.sh filename.pdb script.leap
```

7. Three files will be generated:
- xx113_LEaP.pdb (you can delete this)
- xx113_LEaP_trimmed.pdb
- xx113_LEaP.prmtop

8. Change pdb and prmtop filenames to your preference

9. Done!

NOTES:
- Some CHARMM36-based pdbs may show bond length issues during conversion with CHARMM2AMBER. Running a few steps of energy minimization on the system before conversion should resolve the problem.
- In AMBER/CONVERTED_SYSTEMS you will find two empty directories (ff14SB and ff19SB) where you can store the respective final pdb & prmtop files.

This protocol was tested on:
- Ubuntu 22.04.5 LTS
- Python 3.10.12
- AmberTools 23
- VMD 1.9.3
