# Supplementary material for: Comparison among Amber-ff14SB, Amber-ff19SB, and CHARMM36 Force Fields for Ionic and Electroosmotic Flows in Biological Nanopores
Source: J Chem Theory Comput. 2025 Nov 13;21(22):11772–82. doi: 10.1021/acs.jctc.5c01032 (PMC12659023; doi:10.1021/acs.jctc.5c01032)
Supplement: Supplementary file 2 [file ct5c01032_si_002.pdf]

Supporting Information for: Comparison among Amber ff14SB, Amber ff19SB and CHARMM36 force fields for ionic and electroosmotic flows in biological nanopores

Simone Gargano[a], Domingo Francesco Iacoviello[a], Federico Iacovelli[b], Blasco Morozzo della Rocca[b], Mauro Chinappi[a] *

[a]*Department of Industrial Engineering, University of Rome Tor Vergata, Via del Politecnico 1, 00133, Rome and*
[b]*Department of Biology, University of Tor Vergata, Via della Ricerca Scientifica 1, 00133, Rome*

Supplementary Note S1: Details on simulation set-up and force fields p. S-2

Supplementary Figure S1: I/V Curves for bulk water box at KCl 2M p. S-3
Supplementary Figure S2: CytK sequences and charges exposed toward the lumen p. S-4
Supplementary Figure S3: MspA sequences and charges exposed toward the lumen p. S-5
Supplementary Figure S4: Effect of CUFIX on Amber ff14SB and CytK nanopores p. S-6
Supplementary Figure S5: Effect of CUFIX on Amber ff14SB and MspA nanopores p. S-7
Supplementary Figure S6: Additional data for inter- and intra-variability of ion density p. S-8
Supplementary Figure S7: Average ion distributions at $\Delta V = +125$ mV p. S-9
Supplementary Figure S8: Average ion distributions at $\Delta V = -125$ mV p. S-10
Supplementary Figure S9: RMSD analysis of structural variability in nanopore ROIs p. S-11

Supplementary Table S1: Lipid membrane data for different force fields p. S-12
Supplementary Table S2: Experimental data from literature for CytK and MspA p. S-13
Supplementary Table S3: Summary of numerical conductances p. S-13
Supplementary Table S4: Selectivities from MD simulations p. S-14

Supplementary references: p. S-15

* mauro.chinappi@uniroma2.it

SUPPLEMENTARY NOTE S1: DETAILS ON THE FORCE FIELD USED

In this section, we describe some details of the protocol used to generate the input files for the CHARMM36, Amber-ff14SB and Amber-ff19SB simulations. A directory containing all the relevant scripts is provided as Supporting Information (GARGANO25_CHARMM2AMBER).

A. CHARMM36: topology and parameter files

The July 2018 update of the `c36toppar` topology and parameter files was used to generate all CHARMM36-based [S1] simulation systems, and subsequently to configure the MD simulations of these systems. The files are currently available on `https://mackerell.umaryland.edu/charmm_ff.shtml` (`toppar_c36_jul18.tgz`). Specifically, the files `top_all36_prot.rtf`, `top_all36_na.rtf` and `top_all36_cgenff.rtf` were read by a custom TCL script (`membuild.tcl`) used for generating each simulation system, starting from the standalone protein pdb file to obtain a complete setup comprising the protein (with any required mutations), membrane, water and ions. Parameter files from `toppar_c36_jul18.tgz` were integrated into the NAMD2 [S2] configuration scripts for the execution of the MD simulations. The original `toppar_water_ions.str` file was replaced by the `toppar_water_ions_cufix.str` file provided by `http://bionano.physics.illinois.edu/CUFIX` to include CUFIX [S3] corrections.

B. Generating files for Amber-ff14SB simulations

A custom BASH script (`charmm2amber.sh`) was used to convert pdb files originally used in CHARMM36 simulations (in our case the output of the `membuild.tcl` script discussed above) to generate pdb files formatted for the Amber-ff14SB [S4] force field and the corresponding prmtop files. This script requires the AmberTools23 [S5] package and its *charmmlipid2amber*, *pdb4amber* and *tleap* plugins. All topology and parameter inputs required to generate Amber-based simulation systems are contained in specific files accessed via *leaprc* libraries. In this case, `leaprc.protein.ff14SB`, `leaprc.lipid21`, and `leaprc.water.tip3p` were employed. In particular, TIP3P water [S6] with Joung/Cheatham parameters [S7] for K^+ and Cl^- ions was used. CUFIX corrections for Amber-ff14SB were applied by implementing the `frcmod.ff99cufix` parameter file in the conversion script. This file is available on `http://bionano.physics.illinois.edu/CUFIX`.

C. Generating files for Amber-ff19SB simulations

The same conversion process used for Amber-ff14SB was also implemented to convert CHARMM36-formatted pdb files into pdb files formatted for Amber-ff19SB [S8] and its corresponding prmtop files. All topology and parameter data for Amber-ff19SB were accessed and implemented via `leaprc.protein.ff19SB`, `leaprc.lipid21`, and `leaprc.water.opc`. In particular, OPC water [S9] with Sengupta/Li/Merz parameters [S10] for K^+ and Cl^- ions was used. For certain CHARMM-formatted pdb files (i.e. CytK-6K), an additional energy minimization step could be necessary before conversion with *charmm2amber*, due to bond-length issues that could otherwise affect the conversion process.

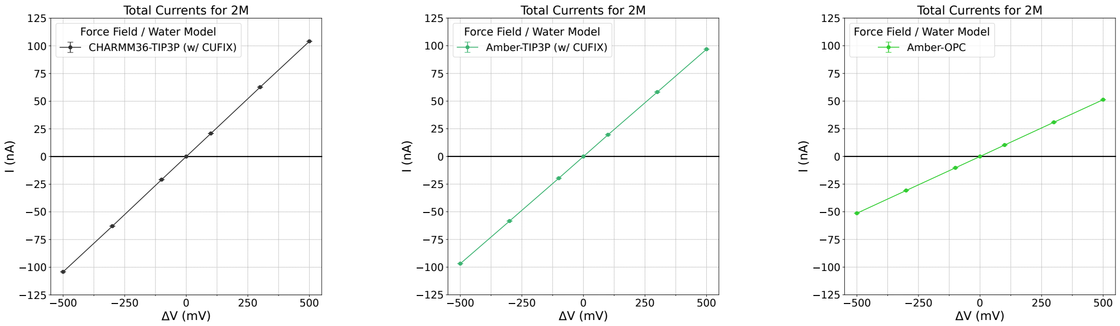

FIG. S1. **I/V Curves for a bulk water box at 2M KCl**. Current-voltage curves for the three cases CHARMM36-TIP3P (w/ CUFIX corrections), Amber-TIP3P (w/ CUFIX corrections) and Amber-OPC. The average conductivity reported in Fig. 2 of the manuscript was calculated as follows. For each voltage, we calculated the average current I and its error using block average, obtaining the I/V curves reported here. Then, we divided each current I by the respective voltage ΔV, obtaining an estimate of the conductance. To estimate the error in the conductance, for each voltage, we first calculated the relative error in the current I and then we multiplied this error by the respective conductance value. We then calculated a weighted average conductance using the errors on conductance estimates for each ΔV as weights. Given the conductance G, the conductivity was calculated as $\sigma = G\frac{L_z}{L_x L_y}$ where L_x, L_y, L_z are the lengths of the periodic water box in the three directions (with the electric field along z). Conductivity error was calculated by multiplying the relative error in the conductance by the conductivity value.

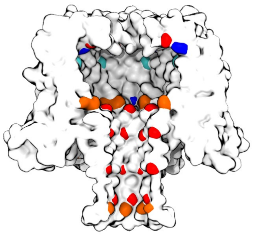

Cytk-2E-4D:

SQVVTDIGQNAKTHTSYNTFNNEQADNMTMSLKVTFIDDP
SADKQIAVINTTGSFMKANPTLSDAPVDGYPIPGASVTLR
YPSQYDIAMNLQDNTSRFFHVAPTNAVEETTVTSSVSYQL
GGSI**D**ASVTPSGPSGESGATG**D**VTWSD**D**VSY**D**QTSYKTNL
IDQTNKHVKWNVFFNGYNNQNWGIYTRDSYHALYGNQLFM
YSRTYPHETDARGNLVPMNDLPTLTNSGFSPGMIAVVISE
KDTEQSSIQVAYTKHADDYTLRPGFTFGTGNWVGNNIKDV
DQKTFNKSFVLDWKNKKLVE

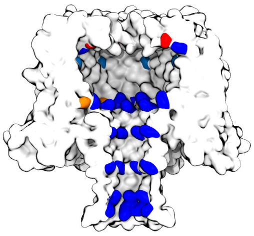

Cytk-6K:

SQVVTDIGQNAKTHTSYNTFNNEQADNMTMSLKVTFIDDP
SADKQIAVINTTGSFMKANPTLSDAPVDGYPIPGASVTLR
YPSQYDIAMNLQDNTSRFFHVAPTNAVE**K**TTVTSSVSYQL
GGSIKASVTPSGPSG**K**SGATG**K**VTWSD**K**VSYKQTSYKTNL
IDQTNKHVKWNVFFNGYNNQNWGIYTRDSYHALYGNQLFM
YSRTYPHETDARGNLVPMNDLPTLTNSGFSPGMIAVVISE
KDTEQSSIQVAYTKHADDYTLRPGFTFGTGNWVGNNIKDV
DQKTFNKSFVLDWKNKKLVE

FIG. S2. **CytK sequences and charges exposed toward the lumen.** All the positive and negative residues exposed toward the lumen are highlighted: negative residues are in red (Asp, D) and orange (Glu, E); while positive residues are in blue (Lys, K). Arginines are not exposed toward the lumen. N-δ-protonated histidine (His, H) is in cyan. This visualization clearly shows that the two pores are highly charged. As shown in Fig. 3 of the manuscript, this strongly influences the selectivity and the EOF. The amino acid sequences of the two nanopores are shown. The mutations with respect to CytK-WT are in bold (CytK-2E4D: K128D-Q145D-S151D-K155D, CytK-6K: 112K-E139K-Q145K-S151K). Both nanopores are missing the first 3 residues from the original CytK-WT sequence and, for this reason, each residue position must be scaled accordingly. The missing residues are not expected to affect the simulation results since the N-terminus is not in the pore lumen.

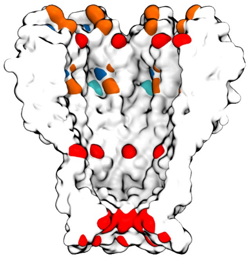

MspA-WT:

GLDNELSLVDGQDRTLTVQQWDTFLNGVFPLDRNRLTREW
FHSGRAKYIVAGPGADEFEGTLELGYQIGFPWSLGVGINF
SYTTPNILIDDGDITAPPFGLNSVITPNLFPGVSISADLG
NGPGIQEVATFSVDVSGAEGGVAVSNAHGTVTGAAGGVLL
RPFARLIASTGDSVTTYGEPWNMN

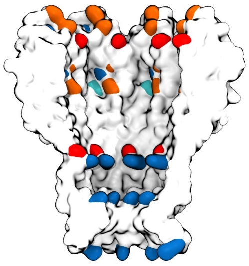

MspA-M3:

GLDNELSLVDGQDRTLTVQQWDTFLNGVFPLDRNRLTREW
FHSGRAKYIVAGPGADEFEGTLELGYQIGFPWSLGVGINF
SYTTPNI**R**I**NN**G**N**IT**R**PPFGLNSVITPNLFPGVSI**R**ADLG
NGPGIQEVATFSVDVSGAEGGVAVSNAHGTVTGAAGGVLL
RPFARLIASTGDSVTTYGEPWNMN

FIG. S3. **MspA sequences and charges exposed toward the lumen.** All the positive and negative residues exposed toward the lumen are highlighted: negative residues are in red (Asp, D) and orange (Glu, E); while positive residues are in blue (Arg, R). Lysines are not exposed toward the lumen. N-δ-protonated histidine (His, H) is in cyan. This visualization clearly shows that both pores are highly charged. As shown in Fig. 4 of the manuscript, this feature strongly affects the selectivity and the EOF. The amino acid sequence of the two nanopores are shown. For MspA-M3, the mutations with respect to MspA-WT are in bold (L88R-D90N-D91N-D93N-A96R-S116R).

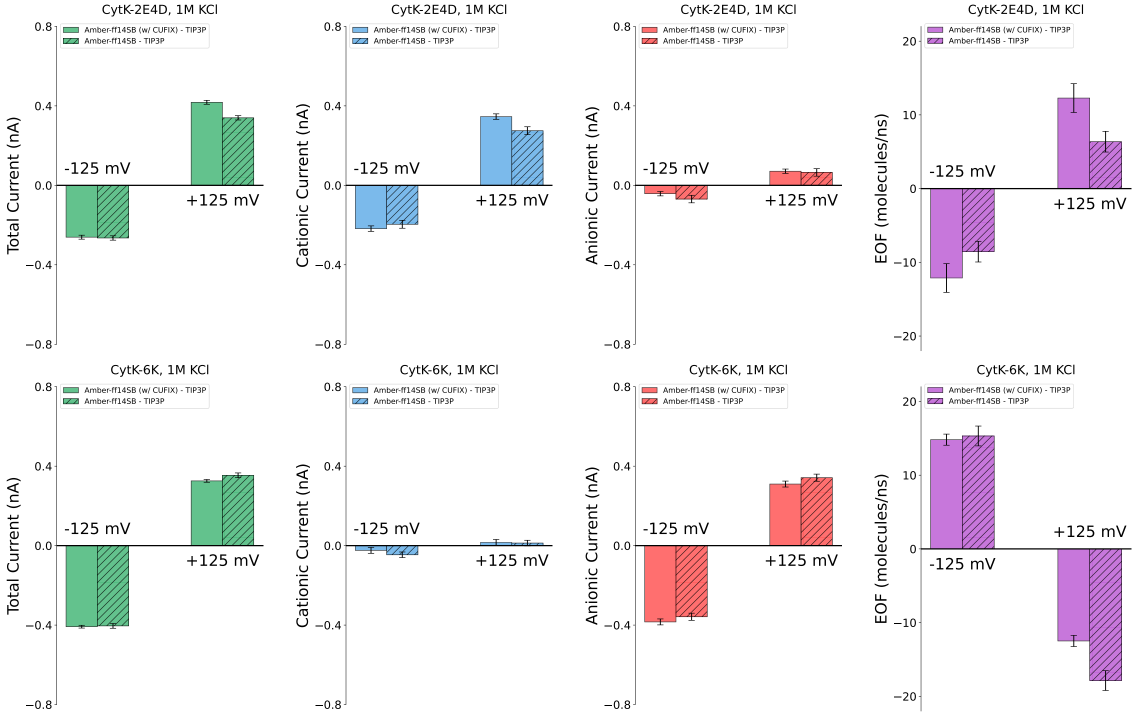

FIG. S4. **Effect of CUFIX corrections on Amber ff14SB and CytK nanopores.** CUFIX corrections (present in the plain bars and absent in the diagonally shaded ones) for ions [S3] have a minor effect on currents and EOF. Qualitatively, as expected, the direction of the EOF is consistent with the nanopore selectivity, *e.g.* for the anion-selective nanopore CytK-6K, the flow has the direction of the negative ions. However, some minor differences are evident for EOF, although the relatively large error bars do not allow us to draw strong conclusions. CUFIX data were already reported in Fig. 3 of the manuscript. As for Fig. 3 of the manuscript, each data point is obtained averaging ten independent non-equilibrium simulations (where equilibration is also repeated for each replica) and the error bars refer to standard errors calculated as the standard deviation divided by $\sqrt{N-1}$ with $N = 10$ the number of replicas. Concerning bulk conductivity σ, the differences between Amber-ff14SB with TIP3P water, with or without CUFIX, are less than 5% for all the concentrations explored in Fig. 2 of the manuscript.

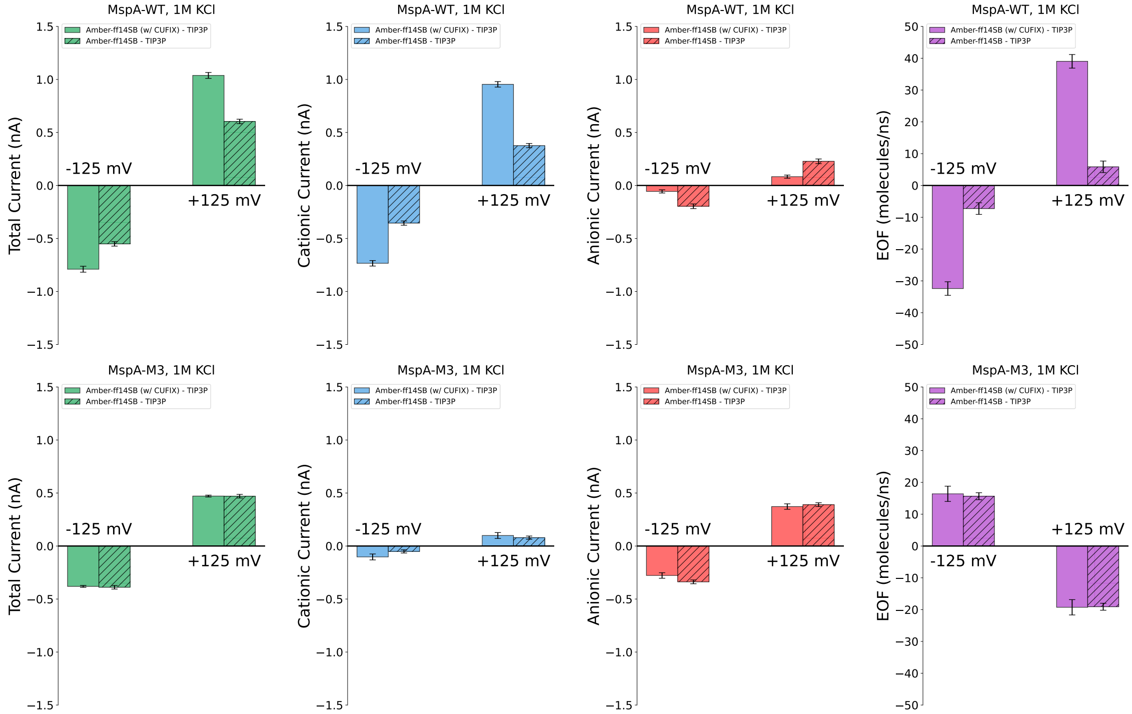

FIG. S5. **Effect of CUFIX corrections on Amber ff14SB and MspA nanopores.** CUFIX corrections (present in the plain bars and absent in the diagonally shaded ones) for ions [S3] significantly affect the ionic currents and EOF for MspA-WT (cation selective). Qualitatively, as expected, the direction of the EOF is consistent with that of the nanopore selectivity, *e.g.* for the cation-selective nanopore MspA-WT, the flow has the direction of the positive ions but CUFIX simulations result in much larger currents. The MspA-M3 data with and without CUFIX are, instead, indistinguishable. CUFIX data had already been reported in Fig. 4 of the manuscript. As for Fig. 4 of the manuscript, each data point is obtained by averaging ten independent non-equilibrium simulations (where also equilibration is repeated for each replica) and the error bars refer to standard errors calculated as the standard deviation divided by $\sqrt{N-1}$ where $N = 10$ is the number of replicas. Concerning bulk conductivity σ, the differences between Amber-ff14SB with TIP3P water, with or without CUFIX, are less than 5% for all the concentrations explored in Fig. 2 of the manuscript.

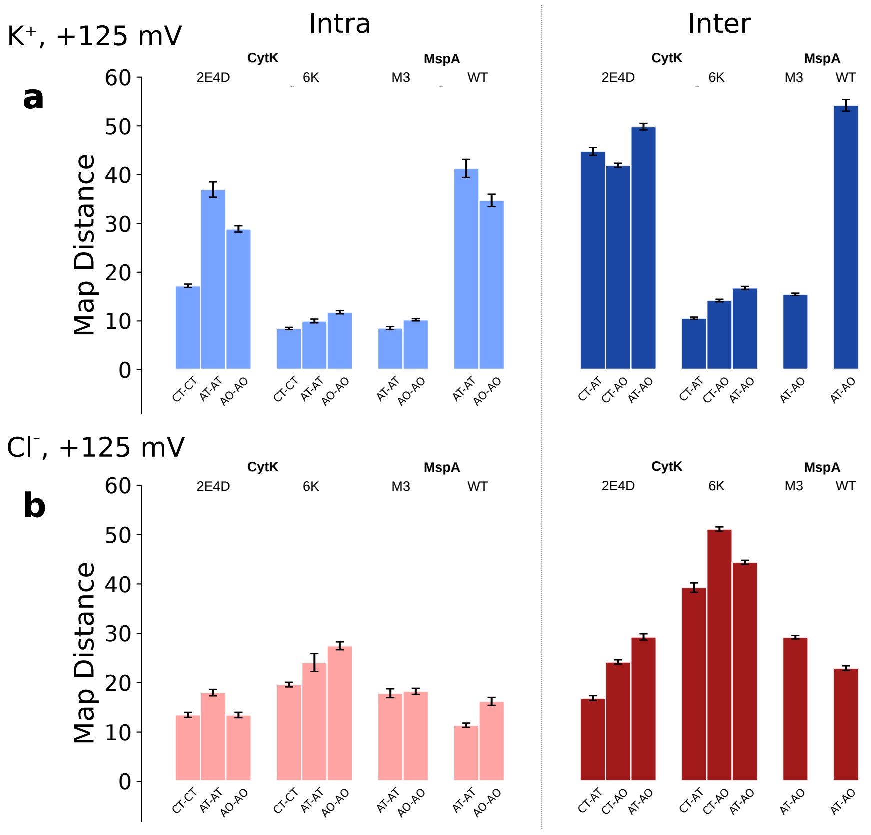

FIG. S6. **Additional data for inter- and intra-variability of ion density distributions.** This figure reports complementary data relative to Fig. 6 of the manuscript. Average distance of Cl^- density maps at $\Delta V = 125mV$. The distance between two force fields is calculated according to Eq. (3) of the manuscript, the results are then averaged. Each bar represents the comparison between two sets. For instance, the bar labeled as CT-CT corresponds to the average of all-against-all comparisons between the 10 CHARMM36 with TIP3P water (45 comparisons) while AO-CT refers to the comparisons among the 10 CHARMM36 with TIP3P and Amber-ff19SB with OPC water (100 comparisons). Errors are calculated dividing the standard deviation by the square root of the number of comparisons. **b** Same analysis for K^+.

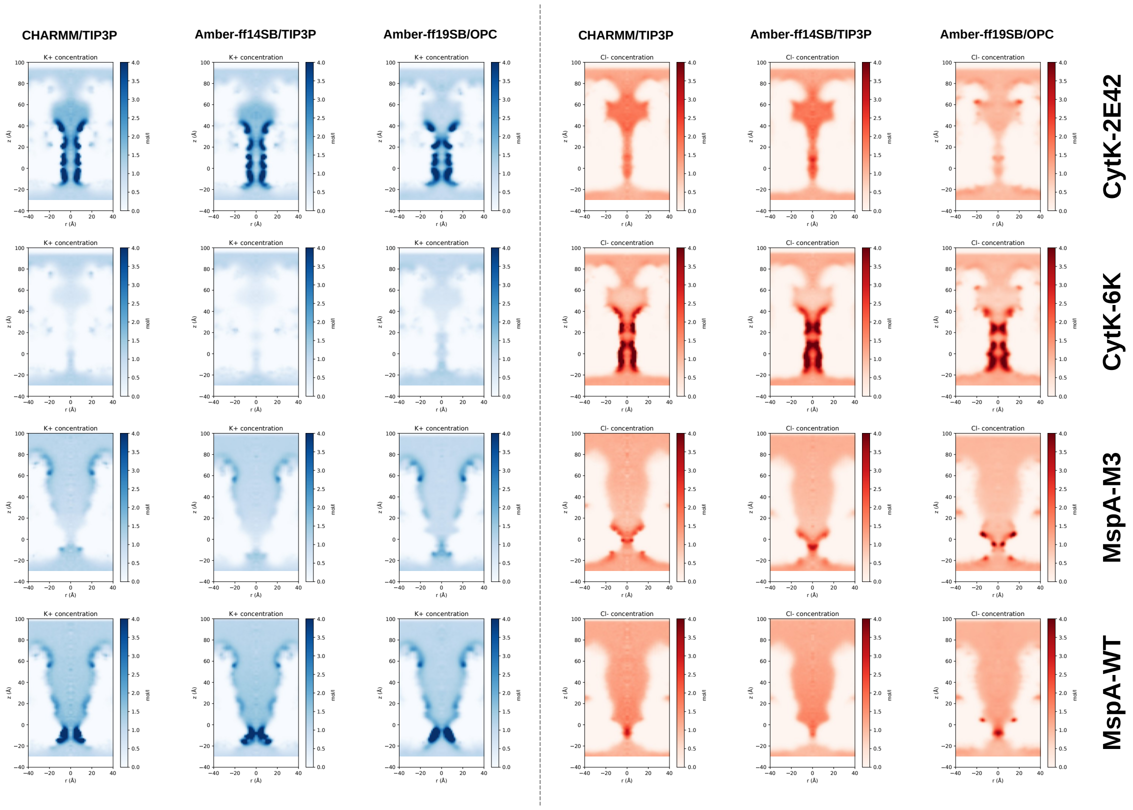

FIG. S7. **Average ion distributions at ΔV = 125 mV.** Ion density distribution maps obtained by averaging the maps from all replicas of each case. Each row of maps corresponds to the system annotated on the right.

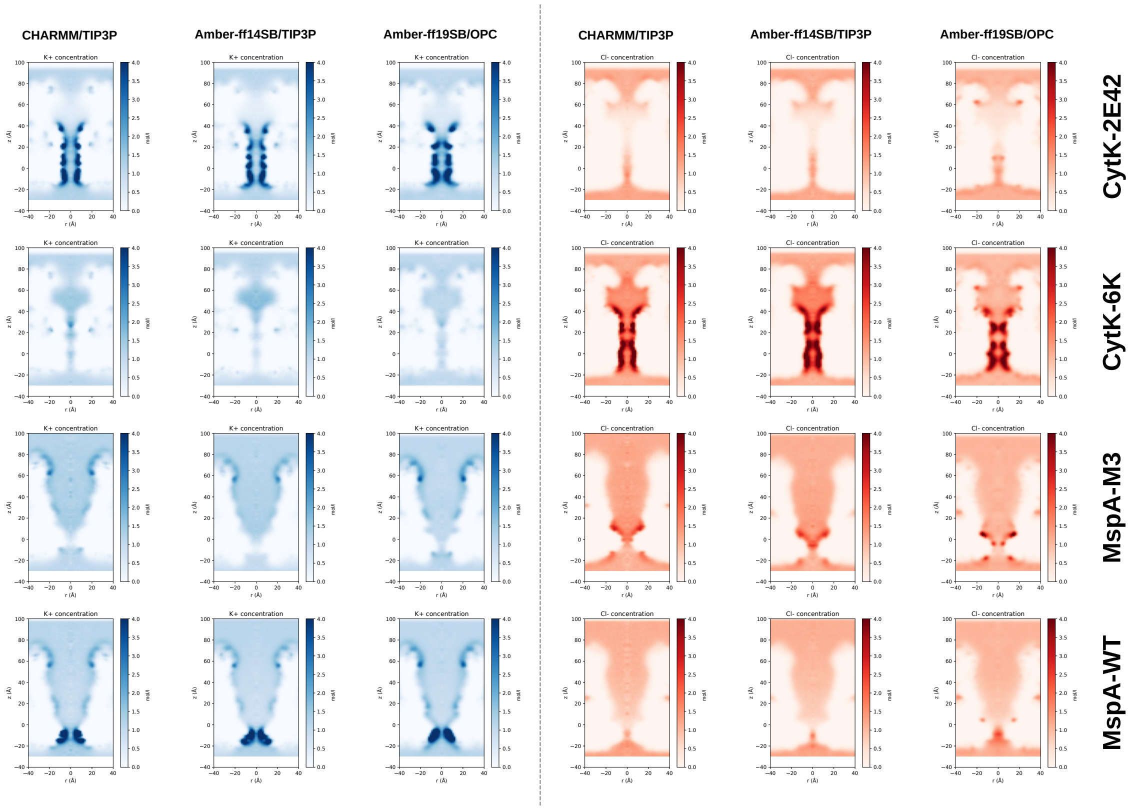

FIG. S8. **Average ion distributions at ΔV = -125 mV.** Ion density distribution maps obtained by averaging the maps from all replicas of each case. Each row of maps corresponds to the system annotated on the right.

—

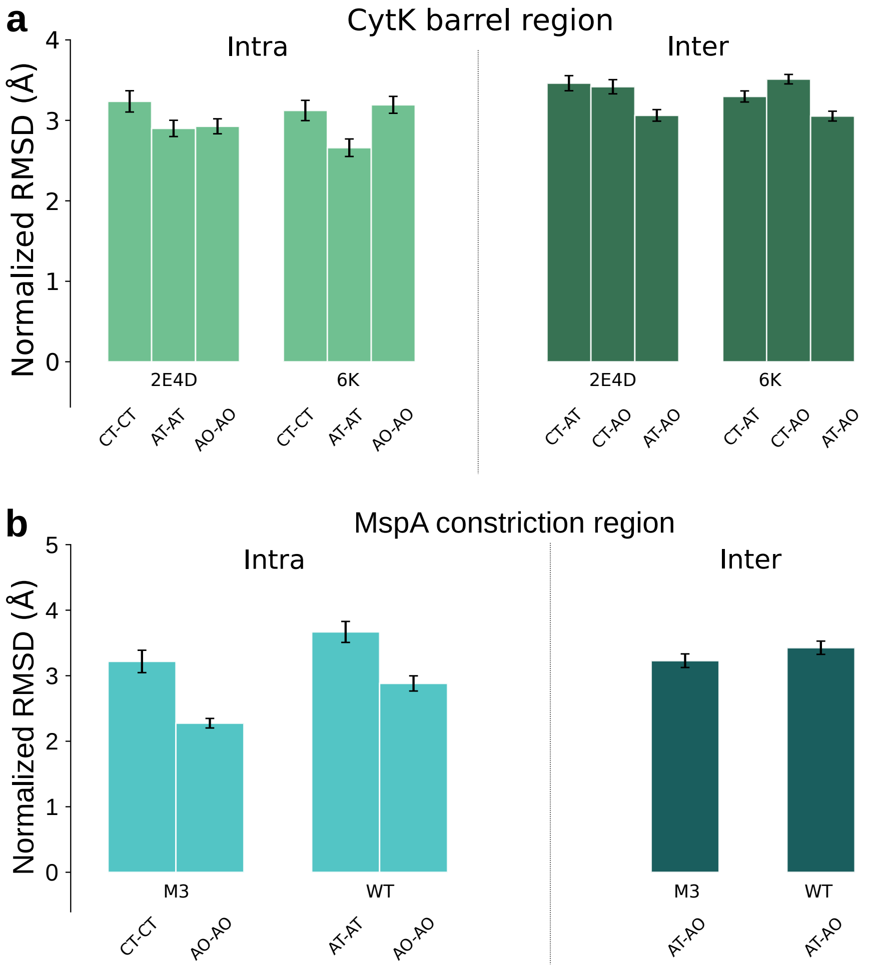

FIG. S9. **RMSD analysis of structural variability in CytK and MspA ROIs.** Root-mean-square deviation (RMSD) values were calculated to evaluate structural variability within specific regions of interest (ROIs) across simulations. Panel (a) shows the average RMSD computed for the β-barrel region of CytK (residues 110–120 and 140–150), while panel (b) shows the same analysis for the constriction region of MspA (residues 89–92, 104–105). Each bar represents the mean RMSD from all pairwise comparisons between two sets of simulations. For instance, the label CT–CT corresponds to the 45 pairwise comparisons among the 10 CHARMM36/TIP3P simulations, whereas AO–CT refers to the 100 cross-comparisons between CHARMM36/TIP3P and Amber-ff19SB/OPC. Error bars represent the standard error of the mean, calculated as the standard deviation divided by the square root of the number of comparisons.

**Nanopore**	**Force Field**	L_x **[Å]**	N_c	N_t	APL_c **[Å^2]**	APL_t **[Å^2]**	**thickness [Å]**
CytK-2E4D	CT	136.48 ±0.20	174.20 ±0.73	180.00 ±1.08	61.87 ±0.37	59.88 ±0.32	39.49 ±0.14
CytK-2E4D	AT	137.72 ±0.20	175.40 ±0.69	181.30 ±0.97	63.39 ±0.43	61.33 ±0.27	38.08 ±0.13
CytK-2E4D	AO	137.91 ±0.24	178.70 ±0.74	181.50 ±1.23	62.52 ±0.48	61.54 ±0.23	37.92 ±0.18
CytK-6K	CT	136.34 ±0.16	174.40 ±0.96	179.50 ±0.59	61.58 ±0.30	59.83 ±0.27	39.57 ±0.12
CytK-6K	AT	136.65 ±0.21	175.60 ±0.53	178.60 ±1.22	61.63 ±0.29	60.61 ±0.38	38.64 ±0.16
CytK-6K	AO	137.33 ±0.14	177.80 ±0.75	180.80 ±1.06	61.92 ±0.40	60.90 ±0.29	38.33 ±0.09
MspA-WT	CT	128.57 ±0.04	143.24 ±0.16	132.18 ±0.28	60.60 ±0.08	65.68 ±0.10	38.53 ±0.02
MspA-WT	AT	129.08 ±0.22	140.70 ±0.80	135.20 ±1.13	62.63 ±0.38	65.19 ±0.38	38.90 ±0.14
MspA-WT	AO	129.97 ±0.19	141.70 ±0.96	138.00 ±0.92	63.84 ±0.52	65.54 ±0.43	37.48 ±0.17
MspA-M3	CT	126.51 ±0.02	139.72 ±0.16	130.53 ±0.16	58.37 ±0.08	62.48 ±0.07	39.25 ±0.02
MspA-M3	AT	129.78 ±0.24	142.20 ±1.36	135.10 ±0.82	63.28 ±0.53	66.58 ±0.37	38.76 ±0.20
MspA-M3	AO	130.54 ±0.15	144.90 ±0.79	137.80 ±0.56	63.44 ±0.30	66.71 ±0.43	37.11 ±0.13

TABLE S1. **Lipid membrane data for the different force fields.** Membrane analysis was performed on systems simulated with different force fields: CHARMM36 (CT), Amber-ff14SB (AT), and Amber-ff19SB (AO). We selected the conformation after the three-step NPT equilibration described in the Methods section of the manuscript. Specifically, we used the .psf and .coor files for CHARMM36 and the .prmtop and .coor files for Amber (ff14SB and ff19SB) simulations. For each configuration, we defined a region of interest comprising the entire box in the x and y directions, excluding a cylindrical region with a radius of 50 Å whose axis coincides with the nanopore axis. In this region of interest, the lipids are sufficiently far from the nanopores and, consequently, their distribution is expected not to be strongly altered by the pore. The number of lipids in the cis and trans leaflets was determined by counting the phosphate head groups in the region of interest. We calculated the area per lipid (APL) for each leaflet by dividing the area of the region of interest by the corresponding number of lipid. Membrane thickness was measured as the distance between the average positions of the phosphate head groups in the upper and lower leaflets. For both CytK and MspA nanopores, the x-axis (L_x) and y-axis (L_y) are identical; consequently, we reported only L_x. The analysis was repeated for each of the 10 independent equilibrations. The table reports the average over the replicas, with errors estimated as the standard error of the mean (standard deviation divided by the square root of the number of replicas). A slightly different protocol was used for the MspA-WT and MspA-M3 runs with CT. In this case, we ran a single equilibration. Consequently, averages were computed over the frames of the final simulation step and, as expected, the confidence intervals are smaller compared to the other cases. The number of lipids differs slightly between the two leaflets. For CytK, the upper leaflet hosts a greater number of lipids compared to the lower leaflet, while the opposite trend is observed for MspA. This is presumably due to the pore asymmetries and the fact that the region of interest is not far enough from the pore. The area per lipid (APL) obtained with the Amber force fields was (in some cases) slightly larger than that obtained with CHARMM36, although both sets are consistent with previous studies [S11, S12]. Conversely, the membrane thickness was slightly greater in the CHARMM36 simulations. Even when these differences are statistically significant (*e.g.* CytK-2E4D for CT and AT), their magnitude is relatively small.

**Nanopore**	**Conductance [nS]**	**Selectivity** P_+/P_-	**Article**	**Source**
MspA-WT	5.1	Faller et al. [S13]	-	Page 3
MspA-WT	4.6	Niederweis et al. [S14]	6.6 ± 0.3	Page 2 / Page 7
MspA-WT	4.1	Trias et al. [S15]	10.4	Table 2 / Page 4
MspA-WT	4.9	Butler et al. [S16]	-	Fig. S1
MspA-M3	2.9	Liu et al. [S17]	-	Page 4
CytK-2E4D	1.5	Sauciuc et al. [S18]	4.04 ± 0.07	Fig. S21 / Fig 1
CytK-6K	1.2	Sauciuc et al. [S18]	0.21 ± 0.08	Fig. S15 / Page 2

TABLE S2. **Experimental data from the literature for CytK and MspA nanopores.** Literature values of single-pore conductance for MspA (both WT and M3) and CytK (2E4D and 6K) nanopores, all measured in 1M KCl. For each entry, we list the bibliographic source and its location in the original work (figure, table, or page). When two fields are reported in the "Source" column, the first refers to the conductance and the second to the selectivity. When the conductance was explicitly reported, it was directly copied. When it was only shown graphically (*e.g.*, I-V curve), the conductance was calculated from the slope of the linear region of the curve.

**Nanopore**	$G_{\mathbf{CT}}$	$G_{\mathbf{AT}}$	$G_{\mathbf{AT\ cufix}}$	$G_{\mathbf{AO}}$
MspA-WT	7.04 ± 0.15	4.62 ± 0.11	7.31 ± 0.13	2.84 ± 0.08
MspA-M3	3.88 ± 0.14	3.44 ± 0.08	3.41 ± 0.10	1.49 ± 0.05
CytK-2E4D	2.83 ± 0.06	2.42 ± 0.06	2.72 ± 0.10	1.18 ± 0.04
CytK-6K	2.20 ± 0.06	3.03 ± 0.07	2.94 ± 0.05	0.90 ± 0.03

TABLE S3. **Summary of conductances for CytK and MspA from our simulations.** Ionic conductances (nS) of MspA (WT and M3) and CytK (2E4D and 6K) nanopores obtained from our simulations using different force fields. G_{CT} refers to CHARMM36 with TIP3P water, G_{AT} to AMBER with TIP3P, $G_{\mathrm{AT\ cufix}}$ to AMBER with TIP3P including the CUFIX corrections, and G_{AO} to AMBER with OPC water. Conductances were obtained by averaging the total ionic current values measured at ±125 mV (Fig. 3 of the manuscript for CytK and Fig. 4 for MspA).

**Nanopore**	I^+/I^-_{CT}	I^+/I^-_{AT}	$I^+/I^-_{\mathrm{AT\ cufix}}$	I^+/I^-_{AO}	Δ_{CT}	Δ_{AT}	$\Delta_{\mathrm{AT\ cufix}}$	Δ_{AO}
MspA-WT	41 ± 97	1.8 ± 0.1	13.1 ± 2.8	4.8 ± 1.6	0.76 ± 0.07	0.16 ± 0.02	0.68 ± 0.02	0.22 ± 0.02
MspA-M3	0.29 ± 0.12	0.15 ± 0.03	0.37 ± 0.06	1.1 ± 0.2	-0.25 ± 0.06	-0.29 ± 0.01	-0.17 ± 0.03	-0.004 ± 0.015
CytK-2E4D	14.5 ± 7.3	2.8 ± 0.7	5.2 ± 1.7	5.5 ± 3.9	0.24 ± 0.02	0.13 ± 0.02	0.18 ± 0.03	0.09 ± 0.02
CytK-6K	0.20 ± 0.06	0.13 ± 0.06	0.06 ± 0.04	1.6 ± 0.5	-0.19 ± 0.02	-0.31 ± 0.03	+0.36 ± 0.02	-0.03 ± 0.02
MspA-WT	2.5 ± 0.5	1.6 ± 0.2	11.5 ± 2.1	2.3 ± 0.3	0.42 ± 0.07	0.15 ± 0.03	0.87 ± 0.03	0.15 ± 0.02
MspA-M3	0.19 ± 0.11	0.20 ± 0.04	0.27 ± 0.08	0.86 ± 0.21	-0.35 ± 0.07	-0.31 ± 0.02	-0.27 ± 0.04	-0.02 ± 0.02
CytK-2E4D	6.5 ± 1.4	4.2 ± 1.3	4.8 ± 0.8	1.5 ± 0.4	0.31 ± 0.02	0.21 ± 0.03	0.27 ± 0.02	0.04 ± 0.02
CytK-6K	-0.03 ± 0.05	0.04 ± 0.04	0.05 ± 0.05	-0.02 ± 0.08	-0.29 ± 0.03	-0.33 ± 0.02	-0.29 ± 0.02	-0.11 ± 0.01

TABLE S4. **Selectivities from MD simulations.** Selectivities can be quantified in two ways: as the ratio of cationic to anionic currents (a measure directly related to the permeability ratio P_+/P_- estimated from reversal potential experiments) and as the difference (Δ) between cationic and anionic currents. The latter approach is usually preferred for MD simulations, because when one of the two currents is close to zero, error propagation in the the ratio leads to very large relative errors. For completeness, we report both calculations. The first four lines refer to $\Delta V = -125$ mV (in this case, we reported $\Delta = -(I^+ - I^-)$), the second block of four lines refers to $\Delta V = +125$ mV. In both cases, the original data are those reported in Fig. 3 and Fig. 4 of the manuscript.

SUPPLEMENTARY REFERENCES

[S1] Jeffery B Klauda, Richard M Venable, J Alfredo Freites, Joseph W O'Connor, Douglas J Tobias, Carlos Mondragon-Ramirez, Igor Vorobyov, Alexander D MacKerell Jr, and Richard W Pastor. Update of the charmm all-atom additive force field for lipids: validation on six lipid types. The journal of physical chemistry B, 114(23):7830–7843, 2010.

[S2] Mark T Nelson, William Humphrey, Attila Gursoy, Andrew Dalke, Laxmikant V Kalé, Robert D Skeel, and Klaus Schulten. Namd: a parallel, object-oriented molecular dynamics program. The International Journal of Supercomputer Applications and High Performance Computing, 10(4):251–268, 1996.

[S3] Jejoong Yoo and Aleksei Aksimentiev. Improved parametrization of li+, na+, k+, and mg2+ ions for all-atom molecular dynamics simulations of nucleic acid systems. The journal of physical chemistry letters, 3(1):45–50, 2012.

[S4] James A Maier, Carmenza Martinez, Koushik Kasavajhala, Lauren Wickstrom, Kevin E Hauser, and Carlos Simmerling. ff14sb: improving the accuracy of protein side chain and backbone parameters from ff99sb. Journal of chemical theory and computation, 11(8):3696–3713, 2015.

[S5] David A Case, Hasan Metin Aktulga, Kellon Belfon, David S Cerutti, G Andrés Cisneros, Vinícius Wilian D Cruzeiro, Negin Forouzesh, Timothy J Giese, Andreas W Götz, Holger Gohlke, et al. Ambertools. Journal of chemical information and modeling, 63(20):6183–6191, 2023.

[S6] William L Jorgensen, Jayaraman Chandrasekhar, Jeffry D Madura, Roger W Impey, and Michael L Klein. Comparison of simple potential functions for simulating liquid water. The Journal of chemical physics, 79(2):926–935, 1983.

[S7] In Suk Joung and Thomas E Cheatham III. Dalkalietermination of alkali and halide monovalent ion parameters for use in explicitly solvated biomolecular simulations. The journal of physical chemistry B, 112(30):9020–9041, 2008.

[S8] Chuan Tian, Koushik Kasavajhala, Kellon AA Belfon, Lauren Raguette, He Huang, Angela N Migues, John Bickel, Yuzhang Wang, Jorge Pincay, Qin Wu, et al. ff19sb: amino-acid-specific protein backbone parameters trained against quantum mechanics energy surfaces in solution. Journal of chemical theory and computation, 16(1):528–552, 2019.

[S9] Saeed Izadi, Ramu Anandakrishnan, and Alexey V Onufriev. Building water models: a different approach. The journal of physical chemistry letters, 5(21):3863–3871, 2014.

[S10] Arkajyoti Sengupta, Zhen Li, Lin Frank Song, Pengfei Li, and Kenneth M Merz Jr. Parameterization of monovalent ions for the opc3, opc, tip3p-fb, and tip4p-fb water models. Journal of chemical information and modeling, 61(2):869–880, 2021.

[S11] Norbert Kučerka, Stephanie Tristram-Nagle, and John F Nagle. Structure of fully hydrated fluid phase lipid bilayers with monounsaturated chains. The Journal of membrane biology, 208(3):193–202, 2006.

[S12] Yalun Yu and Jeffery B Klauda. Update of the charmm36 united atom chain model for hydrocarbons and phospholipids. The Journal of Physical Chemistry B, 124(31):6797–6812, 2020.

[S13] Michael Faller, Michael Niederweis, and Georg E Schulz. The structure of a mycobacterial outer-membrane channel. Science, 303(5661):1189–1192, 2004.

[S14] Michael Niederweis, Sabine Ehrt, Christian Heinz, Uta KloÈcker, Stefanie Karosi, Kristine M Swiderek, Lee W Riley, and Roland Benz. Cloning of the mspa gene encoding a porin from mycobacterium smegmatis. Molecular microbiology, 33(5):933–945, 1999.

[S15] Joaquim Trias and Roland Benz. Permeability of the cell wall of mycobacterium smegmatis. Molecular microbiology, 14(2):283–290, 1994.

[S16] Tom Z Butler, Mikhail Pavlenok, Ian M Derrington, Michael Niederweis, and Jens H Gundlach. Single-molecule dna detection with an engineered mspa protein nanopore. Proceedings of the National Academy of Sciences, 105(52):20647–20652, 2008.

[S17] Hang Liu, Rongliang Wang, Dejian Gu, Shengwei Tan, Hongwen Wu, and Quanjun Liu. Expression and purification of a novel mycobacterial porin mspa mutant in e. coli. Journal of Nanoscience and Nanotechnology, 17(12):9125–9129, 2017.

[S18] Adina Sauciuc, Blasco Morozzo della Rocca, Matthijs Jonathan Tadema, Mauro Chinappi, and Giovanni Maglia. Translocation of linearized full-length proteins through an engineered nanopore under opposing electrophoretic force. Nature Biotechnology, 42(8):1275–1281, 2024.
